# Population health outcomes in Qatar 1990–2023: a systematic analysis for the Global Burden of Disease Study 2023

**DOI:** 10.1016/j.eclinm.2026.103922

**Published:** 2026-05-18

**Authors:** Abdulqadir J. Nashwan, Hana J. Abukhadijah, Mariam N. Al-Mutawa, Ahmad A. Abujaber, Ameena Jesaimani, Binny Thomas, Abdul Rouf Palli Valapila

**Affiliations:** a Nursing & Midwifery Department, Hamad Medical Corporation, 3050, Doha, Qatar; b Clinical Trials Unit, Medical Care and Research Center, Hamad Medical Corporation, 3050, Doha, Qatar; c Corporate Pharmacy Department, Hamad Medical Corporation, 3050, Doha, Qatar

**Keywords:** GBD, Global burden of disease, DALYs, Mortality, Causes of death, Risk factors, Qatar

## Abstract

**Background:**

Patterns of health and disease in Qatar have shifted substantially over the past three decades, reflecting demographic expansion, economic transition, and significant improvements in health system capacity. A comprehensive, up-to-date evaluation of disease burden is essential to guide priority-setting, resource allocation, and national health planning. However, no prior study has systematically assessed long-term trends in morbidity, mortality, and risk factors in Qatar using the most recent epidemiological data consistent with the Global Burden of Disease (GBD) Study framework. This study addresses this gap by providing a detailed analysis of population health outcomes in Qatar from 1990 to 2023.

**Methods:**

We conducted a secondary analysis of publicly available Global Burden of Disease (GBD) 2023 modelled estimates of mortality, prevalence, incidence, disability-adjusted life-years (DALYs), years of life lost (YLLs), and risk factor–attributable disease burden in Qatar. Data sources included vital registration records, hospital and registry datasets, survey data, and modelled estimates, as appropriate. Changes were examined across three major cause categories: non-communicable diseases (NCDs), communicable, maternal, neonatal, and nutritional (CMNN) diseases, and injuries—over four analytic periods (1990–2000, 2000–2015, 2015–2023, and 1990–2023). Uncertainty intervals (UIs) were derived from 250 posterior draws. The study followed GBD guidelines for reporting and estimation transparency.

**Findings:**

Qatar has achieved substantial progress in reducing the burden of CMNN diseases since 1990, driven by expanded immunisation coverage, improved maternal and neonatal care, and strengthened primary health services. However, the burden of NCDs has risen markedly and now accounts for the majority of mortality and DALYs. The leading contributors to NCD burden in 2023 were ischaemic heart disease, diabetes mellitus, cancer, chronic kidney disease, and neurological disorders. High body-mass index, high fasting plasma glucose, hypertension, dietary risks, and tobacco use were the predominant metabolic and behavioural risk factors. Road traffic injuries remained a leading cause of premature mortality, particularly among young men, reflecting Qatar's predominantly male working-age expatriate population and high rates of occupational and vehicular mobility. COVID-19 temporarily reversed declines in infectious disease burden during 2020–2021, although trends returned to levels consistent with the 2018 pre-pandemic trajectory by 2023.

**Interpretation:**

The epidemiological profile of Qatar is characterised by declining infectious disease burden and rising chronic disease and injury burden, shaped by demographic composition and lifestyle transitions. Addressing the growing impact of NCDs and injuries will require sustained preventive strategies, population-level risk factor reduction, strengthened primary care for chronic disease management, and coordinated multisectoral policy responses targeting diet, physical activity, road safety, and occupational health. These findings provide an evidence base to support national health planning and ongoing progress toward equitable and sustainable population health in Qatar.

**Funding:**

The publication of this study was funded by the Medical Research Center (MRC)at Hamad Medical Corporation (HMC), Doha, Qatar.

**Research in context**

**Evidence before this study**

We searched PubMed, Scopus, Web of Science, and the Global Health Data Exchange for studies published between Jan 1, 1990, and June 30, 2025, using combinations of the terms “Qatar”, “disease burden”, “epidemiological transition”, “non-communicable diseases (NCDs)”, “communicable diseases”, “years of life lost (YLLs)”, and disability-adjusted life-years (DALYs). We identified national studies examining individual diseases (such as diabetes, cardiovascular disease, or cancer) and regional analyses of NCDs in the Gulf states; however, these studies generally focused on a single outcome, short time periods, or specific population groups.

Although previous iterations of the Global Burden of Disease (GBD) Study have provided global and regional estimates, to our knowledge, no publication has comprehensively examined Qatar-specific trends in disease burden across mortality, morbidity, risk factor patterns, and demographic context using the most recent GBD 2023 estimates. Prior analyses have also not fully accounted for Qatar's unique population characteristics, including its predominantly young and male expatriate workforce, nor have they incorporated post-pandemic trends.

**Added value of this study**

This study provides the first national-level analysis of disease burden in Qatar spanning more than three decades and incorporating GBD 2023 estimates. By examining mortality, prevalence, incidence, and DALYs for both non-communicable and communicable diseases, as well as injuries, we characterise the country's full epidemiological transition. The analysis reflects that Qatar has achieved notable and sustained reductions in the burden of communicable diseases. At the same time, it indicates a growing predominance of non-communicable diseases, particularly ischaemic heart disease, diabetes, cancer, chronic kidney disease, and neurological disorders. The continued contribution of road traffic injuries to premature mortality is also observed, particularly among young men. Furthermore, we identify high body-mass index, high fasting plasma glucose, hypertension, dietary risks, and tobacco use as leading modifiable risk factors, emphasising opportunities for prevention and intervention.

**Implications of all the available evidence**

Qatar has entered a mature phase of the epidemiological transition, in which infectious disease control successes coexist with growing challenges from chronic, lifestyle-related, and injury-related conditions. The findings underscore the need for health policy and service delivery models that prioritise prevention, long-term chronic disease management, and multisectoral approaches addressing food environments, physical activity, occupational health, and transport safety. Strengthening primary care, integrating population health surveillance, and expanding public health interventions targeting obesity and metabolic disorders will be essential to prevent further escalation of non-communicable disease burden. At the same time, injury prevention strategies must remain a national priority, particularly in high-risk sectors of labour and transport. These results provide an evidence base to guide resource allocation, strategic planning, and coordinated cross-sector health policy action in Qatar.

## Introduction

Patterns of health and disease vary across countries due to the evolving, complex, and dynamic interactions among multiple determinants within populations. Therefore, it is imperative to examine and interpret these patterns within the country's specific national context. Qatar provides a well-developed healthcare system that serves both citizens and expatriate residents. Healthcare is delivered through a strong public sector led by Hamad Medical Corporation and Primary Health Care Corporation, complemented by an expanding private sector. Qatari citizens receive highly subsidised or free healthcare services, while expatriates, including migrant workers who constitute a large proportion of the population, can access affordable care through employer-supported health coverage and the national health card system. Expatriate workers have access to primary, specialised, and emergency services within the country. In addition, individuals requiring additional support may benefit from assistance provided by charitable organisations, NGOs, and semi-governmental institutions that help facilitate access to care and social support. Qatar is also implementing a new national health insurance system aimed at further strengthening healthcare access and coverage for expatriate residents through employer-mandated insurance schemes. When expatriate workers experience serious illness, they may continue receiving treatment in Qatar or may choose to return to their home countries for ongoing care, often with employer support in accordance with national labour regulations.

Qatar, in particular, has a demographic profile that diverges from those of most high-income countries. To elaborate, since 1990, Qatar's population has surged more than sixfold, from approximately 440,000 to over 2.9 million in 2023, a growth driven mainly by labour migration. This growth has resulted in placing more than 90% of residents in the 15–64 age group, and is marked by pronounced male predominance.^1^ This rapid demographic expansion has occurred in parallel with an extraordinary economic transformation, in which Qatar has transitioned from a modest economy with a gross domestic product (GDP) per capita of approximately 13,000 in 1990 to being among the world's top ten countries in per capita income, with an estimated US$71 653 in 2025.^2^ Health sector investments have mirrored this trajectory, with Qatar achieving a Universal Health Coverage (UHC) index score of 76 by 2021, surpassing both the global average (68) and the regional average for the Eastern Mediterranean.^3^ These profound shifts underscore the need to reassess Qatar's health burden profile using updated epidemiological data.

The Global Burden of Disease (GBD) Study has enabled country-specific analyses worldwide. Yet to date, no previous study has comprehensively reported the patterns of health and disease in Qatar. This study aims to address this gap by providing a systematic secondary analysis of GBD 2023 health estimates to characterise population health outcomes in Qatar from 1990 to 2023, and to inform the planning and prioritisation of healthcare resources. This manuscript is part of the GBD Collaborator Network and was conducted in alignment with the GBD 2023 Protocol.^4^

## Methods

Data were extracted from GBD 2023 via the Global Health Data Exchange and analysed using secondary analytical methods. The original GBD 2023 analyses were completed using Python versions 3.8.17 and 3.10.4, Stata version 15.1, and R versions 3.5 and 4.2. The statistical code used in GBD 2023 is publicly available online (http://ghdx.healthdata.org/gbd-2023/code). This study complies with the Guidelines for Accurate and Transparent Health Estimates Reporting (refer to Supplementary Appendix S1).

The GBD 2023 estimates for Qatar draw on multiple primary data sources. Key Qatar-specific inputs include: (1) vital registration data from Qatar's Ministry of Public Health (ICD-10-coded cause-of-death), the primary input to CODEm mortality models; (2) the Qatar STEPwise Approach to Surveillance (STEPS) survey data, providing nationally representative risk factor prevalence estimates; (3) hospital discharge data from Hamad Medical Corporation and the National Cancer Registry, contributing inpatient morbidity inputs; and (4) Primary Health Care Corporation (PHCC) administrative statistics. For conditions where Qatar-specific primary data are limited, GBD 2023 applies spatially informed covariate modelling from the North Africa and Middle East super-region. A proportion of estimates are therefore model-derived rather than directly observed.

The GBD 2023 estimation pipeline employs three principal modelling platforms. CODEm (Cause of Death Ensemble Model) generates cause-specific mortality and YLL estimates. DisMod-MR 2.1 (Disease Modelling Meta-Regression) generates incidence, prevalence, YLD, and DALY estimates. Comparative Risk Assessment (CRA) estimates risk factor-attributable burden using population-attributable fractions. Full methodological detail is discussed elsewhere.^5^

We extracted crude all-ages estimates of deaths, incidence, prevalence, and extracted age-standardised years of life lost (YLLs), and disability-adjusted life-years (DALYs) by cause in Qatar from 1990 to 2023 for both sexes. Data sources used to produce GBD 2023 estimates are listed in the GBD 2023 Sources Tool through the Global Health Data Exchange (https://ghdx.healthdata.org/gbd-2023/sources). Inclusion followed the GBD 2023 framework.

Disease burden was quantified using age-standardised DALY rates for the main cause groups: CMNN, NCDs, and injuries, we used age-standardised rates to support comparisons over time after accounting for differences in age structure and population size. We also estimated cause-specific mortality attributable to risk factors at Level 2. The joinpoint regression analysis, percentage change calculations, and annualised rate of change estimates represent our own secondary analyses applied to the publicly available modelled outputs from GBD 2023.

To contextualise Qatar's findings, age-standardised YLL rates for both sexes were benchmarked against five comparator countries selected on the basis of three pre-specified criteria applied simultaneously: (1) World Bank high-income country classification, ensuring health system maturity comparability; (2) geographic and sub-regional diversity across GBD super-regions; and (3) availability of complete GBD 2023 age-standardised YLL estimates disaggregated by sex for all 20 leading causes. Within these criteria, the United Arab Emirates (UAE) and Kuwait were selected as demographically analogous comparators, both Gulf states characterised by large male-predominant expatriate workforces exceeding 80% of total residents, mirroring Qatar's population structure. Singapore was selected as a high-performing, migration-receiving Asian economy with a comparably large non-citizen labour population. Norway and Luxembourg were included as high-income Western European reference benchmarks to contextualise Qatar's epidemiological transition stage relative to mature, demographically stable economies. Comparisons involving Norway and Luxembourg are intended to illustrate divergence from conventional high-income country epidemiological patterns and do not imply structural or demographic equivalence with Qatar. Race and ethnicity data were not collected or stratified in this analysis, as the GBD 2023 framework structures estimates by age, sex, cause, and geographic location only. The resident population of Qatar is highly heterogeneous; limitations arising from this are discussed below.

DALYs measure the overall fatal and non-fatal burden of a disease and are estimated by summing YLLs and years lived with disability (YLDs). YLLs measure the fatal burden by quantifying premature mortality, defined as the difference between an individual's age at death due to a condition and life expectancy at death, while a YLD measures the non-fatal burden of disease by multiplying incident cases of a condition by the disability weight of that condition and case duration (refer to Supplementary Appendix S2 for GBD metrics definitions).

First, we extracted absolute (all-ages) count estimates of incidence, prevalence, and mortality for 371 diseases and injuries, applying the GBD Study's four-level cause hierarchy for selected years that mark major international global health initiative agendas (1990, 2000, 2015, and 2023). At Level 1, all causes are classified into three overarching groups: CMNN diseases, NCDs, and injuries. These are further disaggregated into 22 Level 2 causes, 174 Level 3 causes, and 301 Level 4 causes (refer to Supplementary Appendix S3 for the full hierarchy).

Second, to contextualise the above findings within global health initiatives agendas we calculated the relative change of the extracted estimates as Percentage Change (PC) and Annualised Rate of Change (ARC) for four-year periods: Pre-Millennium Development Goals Era (pre-MDG Era): spanning from 1990 to 2000, MDG era: spanning from 2000 to 2015, Early Sustainable Development Goals Era (SDG era): spanning from 2015 to 2023, and the overall period (1990–2023) covering the full temporal span to understand our standing with Qatar's achievements towards achieving SDG goals.

Then, we evaluated temporal trends in total DALYs for the major disease causes at level 1 using the Joinpoint Regression Program (version 5.4.0.0), developed by the U.S. National Cancer Institute. This analysis identifies the number of segments (joinpoints) in which a statistically significant change occurred. Each segment is determined by the corresponding years and quantified by the Annual Percent Change (APC) for each segment and the Average Annual Percent Change (AAPC) for the whole segment. We used Monte Carlo Permutation tests to determine the optimal number of joinpoints.

Finally, the results were presented as total numbers and as all-age or age-standardised rates per 100,000 population, with 95% uncertainty intervals (UIs). Mean estimates for all other metrics reported represent the mean across 250 draws from the estimate's distribution, with 95% uncertainty intervals (UIs) calculated as the 2.5th and 97.5th percentile values across the draws. To reduce computing time and resources across the estimation process, the number of draws was decreased from 500 in previous GBD iterations to 250 for GBD 2023. According to the GBD 2023 capstone methods,^5^ internal IHME simulations confirmed that this reduction did not materially affect point estimates or 95% uncertainty interval width for the vast majority of causes and locations. However, for conditions with sparse data or high stochastic variability, minor differences in uncertainty interval width may arise relative to prior GBD iterations; direct comparisons of 95% uncertainty intervals between GBD 2023 and earlier iterations should be interpreted with caution.

### Role of the funding source

The funders of this study had no role in study design, data collection, data analysis, data interpretation, or the writing of the report. The corresponding author had full access to the study data and final responsibility for the decision to submit the manuscript for publication.

## Results

### Temporal trends in DALYs for major disease groups

**Fig. 1:**
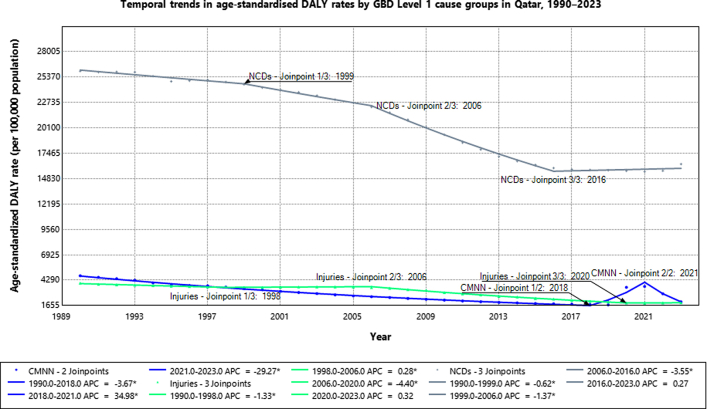
Temporal trends in age-standardised disability-adjusted life-year (DALY) rates by broad cause groups in Qatar (1990–2023).

Qatar has achieved substantial population-health gains since 1990. This is reflected in significant declines in age-standardised DALY rates across major cause groups (Fig. 1). Temporal trends were assessed using age-standardised DALY rates per 100,000 population, which account for Qatar's substantial population growth and changing age structure over the study period. Non-communicable diseases, the dominant contributor of age-standardised DALY rates (ASDR), fell significantly in three consecutive periods: 1990–1999 (APC −0.62%), 1999–2006 (−1.37%), and most sharply 2006–2016 (−3.55%). CMNN causes dropped steadily during 1990–2018 (−3.67%), but were briefly disrupted by a pandemic-era rise in 2018–2021 (+34.98%), then rebounded with a steep decline in 2021–2023 (−29.27%). The 2018 age-standardised CMNN DALY rate serves as the pre-pandemic reference trajectory; by 2023, the rate had returned to levels consistent with this pre-2020 baseline, confirming recovery from the COVID-19-related disruption.

Injuries declined in 1990–1998 (−1.33%), showed a small but significant upward trend in 1998–2006 (+0.28%), and then marked the most important improvements in 2006–2020 (−4.40%). Only statistically significant segments (α = 0.05) are reported. In absolute terms, total NCD DALYs increased from 57,451 (95% UI: 48,842–66,749) in 1990 to 355,891 (289,720–430,008) in 2023, representing a +519.5% increase. Injury DALYs rose from 15,998 (13,838–18,249) to 62,261 (51,391–76,466), a +289.2% increase, while CMNN DALYs increased from 20,434 (17,795–24,544) to 39,415 (31,153–52,292), a +92.9% increase. The shift in risk factor rankings over the study period is illustrated in Supplementary Figure S1, which shows that malnutrition and air pollution dominated DALY-attributable burden in 1990, whereas metabolic risk factors including high body-mass index, high fasting plasma glucose, and dietary risks emerged as the leading contributors by 2023.

### Mortality

Between 1990 and 2023, the deaths from non-communicable diseases (NCDs) in Qatar rose substantially from 714.7 (95% UI: 665.6–757.7) to 2403.4 (2201.4–2611.7), representing a +236.2% increase (202.0%–274.2%). During the SDG era (2015–2023) alone, NCD deaths increased by +43.6% (29.2%–59.6%) with an annualised rate of change (ARC) of +4.6% (3.2%–6.0%). The ARC during this period was the highest compared to earlier decades: +2.9% (1990–2000) and +3.8% (2000–2015). In 2023, the leading causes of NCD mortality were ischaemic heart disease, neoplasms (primarily colorectal and breast cancers), and diabetes-related conditions. A summary of the top 10 causes of mortality, prevalence, and incidence is presented in Table 1, Table 2, Table 3.

**Table 1:** Summary of population health burden in Qatar, 1990 and 2023: top 10 causes of mortality (Panel A).

Rank	Cause	Deaths, 1990	Deaths, 2023	% Change 1990–2023
		Mean (95% UI)	Mean (95% UI)	
Panel A — Top 10 Causes of All-Age Mortality, Qatar (Both Sexes, All Ages)				
1	Ischaemic heart disease	223.4 (178.6–272.5)	**718.9 (592.1**–**846.9)**	**+221.7%**
2	Diabetes mellitus type 2	50.0 (36.3–66.0)	**260.4 (207.5**–**324.2)**	**+420.5%**
3	Road injuries	98.0 (65.7–137.0)	**242.6 (179.2**–**363.6)**	**+147.5%**
4	Chronic kidney disease	39.1 (27.2–53.1)	**166.0 (127.1**–**210.4)**	**+324.5%**
5	Stroke	77.0 (54.4–103.5)	**161.0 (112.9**–**238.2)**	**+108.8%**
6	Lower respiratory infections	38.2 (30.1–48.1)	**113.6 (87.4**–**144.8)**	**+197.1%**
7	Self-harm	20.8 (14.7–29.6)	**90.9 (64.0**–**127.9)**	**+335.4%**
8	Cirrhosis and other chronic liver diseases	41.0 (29.6–54.0)	**80.1 (63.7**–**105.6)**	**+95.1%**
9	Tracheal, bronchus, and lung cancer	18.4 (12.2–26.4)	**79.8 (55.6**–**119.4)**	**+332.1%**
10	Alzheimer's disease and other dementias	12.8 (3.0–34.4)	**76.4 (17.5**–**199.5)**	**+492.7%**

Deaths are absolute all-age counts (both sexes). % change reflects change in absolute counts. Age-standardised mortality rate trends and cause-specific rates per 100,000 are provided in Supplementary Table S1. GBD Level 1 cause groups (NCDs, CMNN, Injuries) and intermediate aggregates are excluded.

All estimates are for both sexes combined, all ages. Values represent mean estimates with 95% uncertainty intervals (UI) derived from 250 GBD 2023 posterior draws. Causes are ranked by absolute 2023 count within each metric. Aggregate and parent-level GBD cause groups are excluded; only specific-level causes are shown. Full cause-level data across all GBD 2023 causes are provided in Supplementary Tables S1–S3. % change reflects change in absolute counts from 1990 to 2023 and should be interpreted in the context of Qatar's > 6-fold population growth over the same period.

GBD, Global Burden of Disease; UI, uncertainty interval; CMNN, communicable, maternal, neonatal, and nutritional; NCD, non-communicable disease; NAFLD, non-alcoholic fatty liver disease; GERD, gastro-oesophageal reflux disease.

**Table 2:** Summary of population health burden in Qatar, 1990 and 2023: top 10 causes of mortality prevalence (Panel B).

Rank	Cause	Prevalent cases, 1990	Prevalent cases, 2023	% Change 1990–2023
		Mean (95% UI)	Mean (95% UI)	
Panel B — Top 10 Causes of All-Age Prevalence, Qatar (Both Sexes, All Ages)				
1	Oral disorders	222,764 (195,715–254,983)	**1,679,229 (1,470,700**–**1,922,880)**	**+653.8%**
2	Cirrhosis and other chronic liver diseases	120,819 (108,374–133,510)	**1,338,867 (1,214,684**–**1,466,456)**	**+1008.1%**
3	Headache disorders	152,360 (134,243–172,129)	**1,186,321 (1,054,016**–**1,340,893)**	**+678.6%**
4	Haemoglobinopathies and haemolytic anaemias	122,523 (113,791–131,531)	**801,861 (743,335**–**859,131)**	**+554.4%**
5	Gynaecological diseases	59,312 (54,213–63,992)	**509,993 (469,882**–**544,652)**	**+759.8%**
6	Diabetes mellitus type 2	26,504 (23,401–29,799)	**445,092 (398,856**–**491,550)**	**+1579.3%**
7	Upper digestive system diseases	50,989 (43,650–60,801)	**439,976 (378,840**–**525,582)**	**+762.8%**
8	Falls	37,237 (31,803–44,151)	**414,529 (349,997**–**498,792)**	**+1013.2%**
9	Age-related and other hearing loss	41,120 (38,168–44,292)	**382,208 (358,359**–**406,624)**	**+829.4%**
10	Tuberculosis	109,756 (93,842–128,097)	**353,529 (300,248**–**417,700)**	**+213.7%**

Prevalent cases are absolute all-age counts (both sexes). Large % change values reflect both true epidemiological change and Qatar's > 6-fold population growth (440,000 in 1990 to ∼2.9 million in 2023). Age-standardised prevalence rates are provided in Supplementary Table S2. Child causes (e.g., caries as subtype of oral disorders; NAFLD as subtype of cirrhosis) are excluded to avoid double-counting.

All estimates are for both sexes combined, all ages. Values represent mean estimates with 95% uncertainty intervals (UI) derived from 250 GBD 2023 posterior draws. Causes are ranked by absolute 2023 count within each metric. Aggregate and parent-level GBD cause groups are excluded; only specific-level causes are shown. Full cause-level data across all GBD 2023 causes are provided in Supplementary Tables S1–S3. % change reflects change in absolute counts from 1990 to 2023 and should be interpreted in the context of Qatar's > 6-fold population growth over the same period.

GBD, Global Burden of Disease; UI, uncertainty interval; CMNN, communicable, maternal, neonatal, and nutritional; NCD, non-communicable disease; NAFLD, non-alcoholic fatty liver disease; GERD, gastro-oesophageal reflux disease.

**Table 3:** Summary of population health burden in Qatar, 1990 and 2023: top 10 causes of mortality incidence (Panel C).

Rank	Cause	Incident cases, 1990	Incident cases, 2023	% Change 1990–2023
		Mean (95% UI)	Mean (95% UI)	
Panel C — Top 10 Causes of All-Age Incidence, Qatar (Both Sexes, All Ages)				
1	Upper respiratory infections	701,059 (613,741–799,214)	**4,408,171 (3,811,604**–**5,088,582)**	**+528.7%**
2	Oral disorders	239,500 (204,531–280,411)	**1,499,265 (1,283,101**–**1,764,225)**	**+525.9%**
3	Diarrhoeal diseases	81,076 (63,250–102,531)	**508,504 (402,655**–**630,512)**	**+527.1%**
4	Gynaecological diseases	45,867 (39,208–53,007)	**400,699 (339,860**–**461,144)**	**+773.6%**
5	Headache disorders	43,170 (36,982–48,948)	**321,813 (278,367**–**370,799)**	**+645.4%**
6	Dermatitis	34,304 (29,702–39,248)	**259,469 (223,000**–**303,360)**	**+656.3%**
7	Urinary diseases and male infertility	28,644 (25,617–32,132)	**224,021 (197,732**–**255,476)**	**+682.0%**
8	Upper digestive system diseases	20,483 (17,461–23,551)	**173,245 (147,530**–**199,926)**	**+745.7%**
9	Falls	19,083 (16,282–22,501)	**146,664 (124,340**–**175,065)**	**+694.6%**
10	Chlamydial infection	21,490 (14,681–30,516)	**144,080 (98,376**–**205,022)**	**+570.4%**

Incident cases are absolute all-age counts (both sexes). COVID-19 is excluded as it has no 1990 baseline and % change is not calculable. Age-standardised incidence rates are provided in Supplementary Table S3. Child causes (e.g., tension-type headache as subtype of headache disorders; GERD as subtype of upper digestive diseases; contact dermatitis as subtype of dermatitis) are excluded.

All estimates are for both sexes combined, all ages. Values represent mean estimates with 95% uncertainty intervals (UI) derived from 250 GBD 2023 posterior draws. Causes are ranked by absolute 2023 count within each metric. Aggregate and parent-level GBD cause groups are excluded; only specific-level causes are shown. Full cause-level data across all GBD 2023 causes are provided in Supplementary Tables S1–S3. % change reflects change in absolute counts from 1990 to 2023 and should be interpreted in the context of Qatar's > 6-fold population growth over the same period.

GBD, Global Burden of Disease; UI, uncertainty interval; CMNN, communicable, maternal, neonatal, and nutritional; NCD, non-communicable disease; NAFLD, non-alcoholic fatty liver disease; GERD, gastro-oesophageal reflux disease.

In comparison, deaths from communicable, maternal, neonatal, and nutritional (CMNN) diseases changed from 195.4 (95% UI: 173.4–220.0) in 1990 to 263.9 (229.5–301.6) in 2023, reflecting a +35.0% increase (12.6%–61.8%). During the SDG era, deaths increased by +30.6% (7.3%–58.9%) with an ARC of +3.3% (0.8%–5.9%). This growth followed an earlier −30.5% decline (−41.6% to −17.4%) from 1990 to 2000 (ARC: −3.6%), and a +48.7% increase (23.1%–79.6%) between 2000 and 2015 (ARC: +2.6%). In 2023, the leading causes of CMNN mortality were respiratory infections and tuberculosis, followed by lower respiratory infections and neonatal disorders. The top 10 causes of mortality in 1990 and 2023 are shown in Table 1 (Panel A). Detailed cause-specific deaths and relative change are shown in Supplementary Table S1.

### Prevalence

Over the past three decades, Qatar has witnessed a significant increase in the prevalence of non-communicable diseases (NCDs). In 1990, approximately 408,358 individuals were affected (95% UI: 404,041–413,265). By 2023, this number had soared to 2,943,098 (2,923,043–2,964,388), marking a +620.7% increase (611.1%–630.3%). This dramatic rise was most rapid between 2000 and 2015, with a +327.3% increase and an ARC of +10.1%. During the SDG era (2015–2023), NCDs prevalence rose at a slower pace of +26.5% (25.1%–28.0%), with an ARC of +2.9%.

In contrast, the prevalence of CMNN disease increased from 252,192 (228,591–279,043) in 1990 to 1,292,822 (1,185,042–1,416,549) in 2023, a +412.6% rise (348.4%–486.0%). Like NCDs, the steepest increase occurred between 2000 and 2015 (+233.9%), with an ARC of +8.3%. During the SDG period, prevalence rose only by +27.2% (11.1%–45.7%), and the ARC was +3.0%.

In 2023, the most prevalent NCD was oral disorders, affecting over 1.67 million people, while among CMNN diseases, respiratory infections and tuberculosis accounted for the highest burden with nearly 599,000 cases. The top 10 prevalent conditions in 1990 and 2023 are shown in Table 2 (Panel B). Detailed prevalences and relative change are shown in Supplementary Table S2.

### Incidence

Over the past three decades, Qatar has experienced a substantial increase in the incidence of non-communicable diseases (NCDs). In 1990, there were 625,888.2 incident cases (95% UI: 587,185.6–670,473.9). By 2023, this reached 4,335,507.7 (4,104,585.8–4,616,176.9), marking a +592.6% increase (533.9%–656.8%). The largest period-specific increase occurred between 2000 and 2015 (+295.0% [262.6%–330.2%]; ARC +9.5% [8.9%–10.2%]). During the SDG era (2015–2023), NCD incidence increased by +29.4% only (19.4%–40.3%), with an ARC of +3.2% (2.2%–4.3%). In 2023, the highest-incidence NCD categories were oral disorders (1,499,264.6 [1,283,101.2–1,764,225.4]), other skin and subcutaneous diseases (459,876.3 [445,117.6–477,724.0]), and neurological disorders (323,877.2 [280,715.5–372,571.7]).

In contrast, the incidence of communicable, maternal, neonatal, and nutritional (CMNN) diseases increased from 913,197.2 (95% UI: 820,754.2–1,017,503.8) in 1990 to 9,901,298.9 (8,401,151.1–11,601,159.0) in 2023, a +984.2% rise (793.1%–1216.2%). The steepest increase occurred between 2000 and 2015 (+288.5% [232.2%–354.4%]; ARC +9.4% [8.3%–10.6%]); during the SDG period, incidence rose by +125.8% (85.4%–175.0%), with an ARC of +10.7% (8.0%–13.4%). In 2023, the highest-incidence CMNN categories were respiratory infections and tuberculosis (8,969,001.0 [7,496,965.3–10,688,189.2]), enteric infections (508,633.2 [402,765.2–630,640.2]), and HIV/AIDS and sexually transmitted infections (246,914.9 [177,055.4–341,120.9]). The top 10 incident conditions in 1990 and 2023 are shown in Table 3 (Panel C). Detailed incidence and relative change are shown in Supplementary Table S3.

### Risk factors

**Fig. 2:**
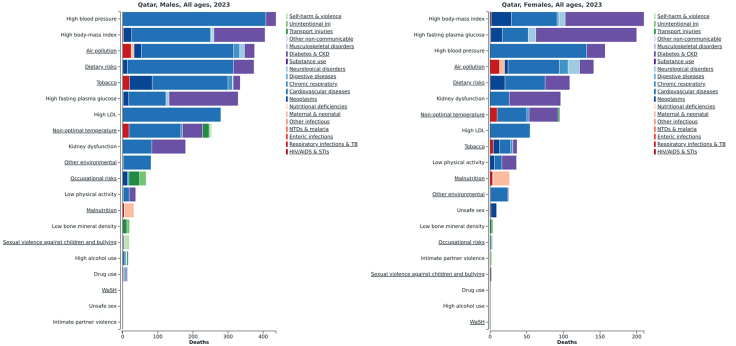
Risk factor–attributable deaths in Qatar, 2023, by sex and cause.

Fig. 2 presents the leading modifiable risk factors contributing to mortality among males and females. In males, the leading risk-attributable deaths arise from high systolic blood pressure, high body mass index (BMI), air pollution, dietary risks, and tobacco. In females, high BMI ranks first, followed by high fasting plasma glucose, high systolic blood pressure, air pollution, and then high low-density lipoprotein (LDL) cholesterol. The stacked segments show that most deaths from cardiovascular diseases (CVD) and diabetes/chronic kidney disease (CKD) are attributable to these risk factors.

### Years of life lost for Qatar and comparator countries

**Fig. 3:**
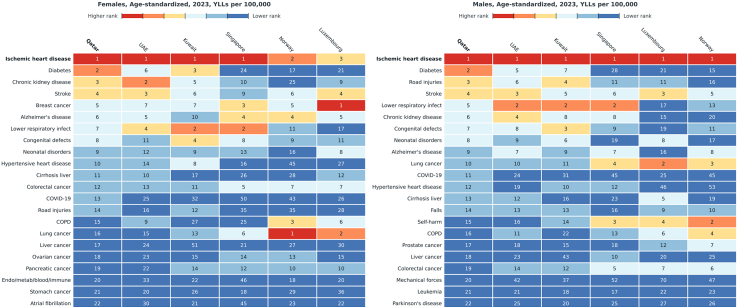
Cross-country comparison of cause-specific YLL rankings in 2023: Qatar and selected countries.

Fig. 3 ranks the leading causes of premature mortality (YLLs per 100,000) for Qatar, UAE, Kuwait, Singapore, Luxembourg, and Norway. Among the 20 leading causes of age-standardised YLLs in 2023, Qatar's top five in males were ischaemic heart disease, diabetes, road injuries, stroke, and lower respiratory infections, a profile broadly comparable to the UAE and Kuwait, but diverging from Singapore, Luxembourg, and Norway, where diabetes and injuries rank much lower. Among females, the top five leading causes were ischaemic heart disease, diabetes, chronic kidney disease, stroke, and breast cancer (Fig. 3). This profile is closer to the UAE and Kuwait and contrasts with Singapore, Luxembourg, and Norway, where diabetes and CKD rank lower.

## Discussion

This study provides the first secondary descriptive and trend analysis of Qatar's national disease burden spanning 33 years using GBD 2023 estimates.

The trend changes identified by the joinpoints coincide temporally with identifiable policy and demographic milestones. For NCDs, the 1999 joinpoint corresponds to Qatar's early National Health Policy and HMC consolidation. The 2006 joinpoint coincides with Qatar National Vision 2030 planning^6^ and rapid urbanisation, both accompanied by mass labour immigration. The 2016 joinpoint aligns with NHS 2011–2016 culmination^7^ and PHCC establishment^8^; the subsequent stabilisation (APC +0.27%) may reflect lifestyle-driven burden outpacing healthcare system gains. For CMNN diseases, the 2018 joinpoint precedes COVID-19; the APC of +34.98% during 2018–2021 captures SARS-CoV-2 importation and pandemic-related disruption, while the APC of −29.27% during 2021–2023 reflects Qatar's rapid vaccine rollout.^9^ These associations are temporal and should not be interpreted as causal attributions.

The findings carry direct implications for Qatar's National Health Strategy 2024–2030.^10^ The emergence of high BMI as the leading risk factor for female mortality underscores the urgency of continuous monitoring by Qatar's National Obesity Prevention Programme (STEPwise Survey 2023: 41.4% adult obesity). The prominence of Type 2 diabetes as the second leading cause of mortality (+420.5% since 1990) supports continued investment in the National Diabetes Programme.^11^ The persistence of road injuries as the third leading cause of male mortality argues for targeted enforcement of Qatar's National Road Safety Strategy (2013–2022). Structural drivers of NCD risk, including limited walkability due to extreme summer heat, the dominance of ultra-processed foods, and sedentary work patterns, require coordinated intersectoral action consistent with the NHS 2024–2030 governance framework.

The decline in CMNN burden is consistent with the epidemiologic transition theory, which describes the movement of societies from a predominance of infectious disease to chronic, degenerative conditions as life expectancy, income, and living standards increase.^12^ In Qatar, this transition has been supported by sustained national investment in maternal and child health services, expanded immunisation coverage, and strengthened primary care and universal health coverage, and improvements in cardiovascular disease outcomes that placed Qatar among the best-performing countries in the Eastern Mediterranean Region^13^, ^14^, ^15^ These developments align with Qatar's attainment of a Universal Health Coverage (UHC) index score that surpasses both global and regional averages, reflecting the maturity of health system infrastructure and accessibility.^3^ Nevertheless, the temporary rise in CMNN-related mortality during 2020–2021 observed in this study corresponds to the global disruption caused by the COVID-19 pandemic. This effect mirrors global patterns, as the pandemic caused a marked increase in the burden of respiratory infections while simultaneously reducing access to routine care for chronic diseases and preventive services.^16^^,^^17^ The subsequent decline in CMNN burden after 2021 may suggest that Qatar's health system rapidly restored essential services and adapted to pandemic-related pressures.

Over the same period, the burden of NCDs has risen markedly and now constitutes the dominant source of mortality and disability in Qatar. The most significant contributors include ischaemic heart disease, type 2 diabetes mellitus, cancer, chronic kidney disease, and neurological disorders. This pattern corresponds closely with trends in other Gulf Cooperation Council (GCC) states undergoing rapid economic development, urbanisation, and lifestyle change.^18^, ^19^, ^20^ The absolute DALY counts presented in the Results section warrant careful contextualisation against Qatar's extraordinary demographic expansion. The +519.5% rise in absolute NCD DALYs, from 57,451 in 1990 to 355,891 in 2023, is broadly proportional to the country's more than sixfold population growth over the same period, indicating that the increase in total NCD burden largely reflects demographic expansion rather than worsening individual-level risk. This interpretation is corroborated by the concurrent decline in age-standardised NCD DALY rates from 1990 to 2016, confirming genuine per-person health improvement. By contrast, CMNN absolute DALYs grew only 1.9-fold and injury absolute DALYs 3.9-fold, both well below the rate of population growth, reflecting true reductions in per-capita communicable disease and injury burden over the study period. The partial reversal of the NCD age-standardised trend post-2016 (APC +0.27%) signals an emerging epidemiological challenge that warrants sustained prevention investment even as absolute burden growth remains primarily demographic in origin. The risk factor profile observed in this study underscores the central role of modifiable metabolic and behavioural factors. High body-mass index, high fasting plasma glucose, hypertension, dietary risks, and tobacco use were the leading contributors to premature mortality, reflecting national and global patterns associated with cardiometabolic disease burden.^5^^,^^21^

Obesity in particular has emerged as one of Qatar's most pressing public health challenges. The prevalence of overweight and obesity among both adults and adolescents ranks among the highest worldwide, driven in part by dietary transitions, sedentary lifestyles, and environmental factors that discourage physical activity.^19^^,^^22^^,^^23^ The downstream consequences of obesity—including type 2 diabetes, cardiovascular disease, and chronic kidney disease—account for a substantial proportion of DALYs in both men and women, reinforcing the urgency of intensifying preventive and lifestyle-focused interventions.

Road traffic injuries, in particular, remain a leading cause of premature mortality and years of life lost in Qatar. Despite advances in emergency response capacity, trauma care, and legislative reforms related to road safety, injury mortality continues to reflect the intersection of demographic composition, high vehicle use, rapid motorisation, and labour-related mobility.^24^^,^^25^ The persistence of road traffic injuries as a major contributor to the health burden underscores the need for sustained, multisectoral prevention strategies that involve transportation planning, law enforcement, occupational regulation, and public education.

Interpretation of health trends in Qatar must also account for the country's unique demographic structure. More than 85% of Qatar's population is between the ages of 15 and 64, with men outnumbering women by more than 3 to 1 due to the predominance of expatriate labour.^1^ This demographic composition influences disease patterning. Occupational and transportation-related injuries occur more frequently among men, who constitute the majority of the manual labour workforce.^1^ Meanwhile, metabolic and obesity-related NCD risks are expressed differently across sexes, with women experiencing a disproportionate burden of obesity and insulin resistance relative to men.^26^ Further complexity arises because many expatriate residents do not remain in Qatar throughout the full progression of chronic illnesses; as a result, long-term morbidity and mortality associated with diseases diagnosed in Qatar may occur outside the country's reporting systems.^27^ Recognising this dynamic is essential for accurate health forecasting and resource planning.

Additionally, Qatar's health landscape is strongly shaped by workforce patterns and occupational exposures among its predominantly expatriate labour population. Previous national assessments and trauma-system evaluations have demonstrated that high-risk labour sectors contribute disproportionately to premature mortality, road injuries, and cardiometabolic risk. Integrating occupational health, injury prevention, and chronic disease management, particularly within high-mobility expatriate populations, will therefore be critical for reducing YLLs and improving population health outcomes.^24^^,^^25^^,^^28^^,^^27^

The findings of this study have clear implications for health policy and system priorities in Qatar. The continuing rise in NCD burden underscores the importance of reorienting healthcare delivery toward preventive, community-based, and integrated chronic disease management. Strengthening primary care remains a pivotal strategy, particularly for early detection of metabolic risk, support for medication adherence, and coordinated long-term follow-up. Promoting healthier dietary practices, expanding opportunities for physical activity, and addressing environmental determinants of lifestyle behaviour will require coordinated action across sectors, including education, food systems, transport, labour, and urban planning. Additionally, improving data interoperability and adopting real-time digital health surveillance will be crucial to monitoring disease trends and the impact of interventions across a mobile, diverse population.

This paper may further inform and refine the policy implications outlined here, particularly in relation to strengthening primary care, enhancing chronic disease management models, and supporting multisectoral strategies to address obesity, metabolic risk, and injury prevention at the population level.

This study also has several limitations. A notable limitation of this study is that GBD estimates for Qatar rely on regional proxies to bridge gaps in national data, a method that may overlook the country's unique health profile.

GBD estimates are partly based on statistical modelling, particularly for conditions where surveillance or registry data are limited. Although the GBD framework employs rigorous, validated methods, some degree of uncertainty is inherent in modelled estimates.^5^ A further methodological consideration concerns the applicability of GBD modelling algorithms to Qatar's uniquely atypical population structure.

GBD 2023 employs CODEm for mortality estimation and DisMod-MR 2.1 for morbidity outcomes, both of which draw on cross-national covariates and Bayesian spatiotemporal priors calibrated to global and regional patterns. For Qatar, where the 15–64 working-age cohort constitutes over 85% of the population and the male-to-female ratio exceeds 3:1, standard age-standardisation procedures may partially attenuate the influence of this atypical structure on disease burden metrics. For conditions with limited national primary data, GBD estimates draw on regional covariate patterns from the North Africa and Middle East super-region, which may not fully reflect Qatar's specific epidemiological environment. Readers are urged to interpret these estimates within this context.

Furthermore, variation in diagnostic coding practices and data completeness across healthcare providers may affect the attribution of cause-specific mortality.^29^ Qatar's highly mobile expatriate population also presents unique epidemiological challenges, as long-term disease outcomes may not be captured if individuals leave the country during later disease stages. Additionally, while the comparative risk assessment framework identifies the proportional contribution of modifiable risk factors, these estimates reflect population-level associations and should not be interpreted as direct causal inference at the individual level.^5^ Furthermore, the transient nature of the expatriate population introduces a distinct statistical bias. Individuals facing life-threatening conditions or chronic illness often choose to return to their home countries for familial support and long-term care. Because these deaths and disabilities occur outside of Qatar, even if the individual is technically still a resident, they are not captured in local morbidity and mortality registries. This systematic ‘out-migration’ of the ill suggests that GBD figures likely underestimate the true longitudinal health burden associated with residency in Qatar.

Moreover, the selection of comparator countries was guided by pre-specified criteria; however, the fixed structure of publicly available GBD 2023 outputs precludes formal sensitivity testing with comparator countries, and the findings of the cross-national comparison should be interpreted within this constraint. Finally, although GBD 2023 incorporates adjustments for pandemic-associated disruptions, the lingering effects of COVID-19 on health-seeking behaviour and service utilisation may continue to influence short-term trends.^9^

Overall, Qatar has achieved substantial improvements in communicable disease control and maternal-child health over the past three decades, reflecting major advancements in healthcare access and system performance. However, the growing dominance of non-communicable diseases, driven primarily by modifiable metabolic and lifestyle risk factors, represents a major public health challenge. Efforts to strengthen preventive health policies, enhance primary care capacity, and implement multisectoral approaches to address the structural drivers of obesity and metabolic disease will be essential to sustaining progress and optimising population health outcomes.

Over the past three decades, Qatar has made substantial progress in reducing the burden of communicable, maternal, neonatal, and nutritional diseases, reflecting sustained investment in healthcare infrastructure, strengthening primary care, and social development. However, the growing predominance of non-communicable diseases and the persistent contribution of road traffic injuries to premature mortality highlight an evolving health landscape shaped by demographic dynamics, lifestyle transitions, and occupational and environmental exposures. Addressing these challenges will require sustained commitment to preventive health strategies, comprehensive risk factor reduction, and integrated models of chronic disease management anchored in strong primary care. Multisectoral action across education, transport, labour, and urban planning will be essential to create health-supportive environments and reduce structural drivers of risk. Continued enhancements to surveillance systems and the timely translation of research into policy will be key to ensuring that Qatar's health system remains responsive, equitable, and positioned to safeguard population health across future generations.

## Contributors

Contributors: Conceptualisation: AJN; data curation: HJA; formal analysis: HJA; funding acquisition: APV and AJ; supervision: AJN; writing—original draft: AJN, HJA, APV, BT, AJ, MNT, and AAA; writing—review & editing: AJN, HJA, APV, BT, AJ, MNT, and AAA. AJN and HJA verified the underlying data. All authors read and approved the final version of the manuscript.

HJA (corresponding author) had full access to all data extracted from the Global Health Data Exchange for this study and takes responsibility for the integrity of the data and the accuracy of the analysis. AJN, as the senior author, independently verified the analytical outputs and interpreted the findings. The decision to submit the manuscript for publication was made by HJA in consultation with AJN.

## Data sharing statement

This study follows the Guidelines for Accurate and Transparent Health Estimates Reporting (GATHER). To download citations and metadata for the input data sources used in the GBD 2023 analyses presented in this study, please visit the GBD 2023 Sources Tool (https://ghdx.healthdata.org/gbd-2023/sources).

## Declaration of interests

The authors declare no competing interests.

## Appendix A Supplementary data

Supplementary Appendix S3

Supplementary Captions

## References

[1] Planning and Statistics Authority (2020). https://www.npc.qa/en/statistics/census2020/Pages/results/default.aspx.

[2] World Bank (2025). GDP per capita (current US$) - Qatar. https://data.worldbank.org/indicator/NY.GDP.PCAP.CD?locations=QA.

[3] World Health Organization, International Bank for Reconstruction and Development/The World Bank (2023). https://www.who.int/publications/i/item/9789240080379.

[4] Institute for Health Metrics and Evaluation (2024). https://www.healthdata.org/research-analysis/about-gbd/protocol.

[5] GBD 2023 Disease and Injury and Risk Factor Collaborators (2025). Burden of 375 diseases and injuries, risk-attributable burden of 88 risk factors, and healthy life expectancy in 204 countries and territories, including 660 subnational locations, 1990-2023: a systematic analysis for the Global Burden of Disease Study 2023. Lancet.

[6] General Secretariat for Development Planning (2008). https://www.gco.gov.qa/en/state-of-qatar/qatar-national-vision-2030/our-story/.

[7] Supreme Council of Health (2011). https://faolex.fao.org/docs/pdf/qat209077E.pdf.

[8] State of Qatar (2012). https://www.phcc.gov.qa/media/news/2023/06/04/phcc-celebrates-its-10-year-anniversary.

[9] AlNuaimi A.A., Chemaitelly H., Semaan S. (2023). All-cause and COVID-19 mortality in Qatar during the COVID-19 pandemic. BMJ Glob Health.

[10] Ministry of Public Health Qatar (2024). https://www.moph.gov.qa/english/NHS/Pages/About.aspx.

[11] Al-Thani M.H.J.T., El-Saharty S., Jamal Z. (2025). Qatar's progress in curbing diabetes: a comprehensive and proactive approach. Health Syst Reform.

[12] Omran A.R. (2005). The epidemiologic transition: a theory of the epidemiology of population change. Milbank Q.

[13] Kandy M.C., Abdulmajeed J., Gohel C.N., Gibb J.M., Al-Kuwari M.G. (2024). Transforming primary healthcare services with centralized health intelligence: a case study from Qatar. Qatar J Public Health.

[14] GBD 2015 Eastern Mediterranean Region Cardiovascular Disease Collaborators (2018). Burden of cardiovascular diseases in the Eastern Mediterranean Region, 1990-2015: findings from the Global Burden of Disease 2015 study. Int J Public Health.

[15] World Health Organization (2025). Country cooperation strategy for WHO and Qatar 2024-2030. Cairo: WHO Regional Office for the Eastern Mediterranean. https://www.who.int/publications/i/item/9789292745448.

[16] COVID-19 Excess Mortality Collaborators (2022). Estimating excess mortality due to the COVID-19 pandemic: a systematic analysis of COVID-19-related mortality, 2020-21. Lancet.

[17] Khatib M.Y., Ananthegowda D.C., Elshafei M.S. (2022). Predictors of mortality and morbidity in critically ill COVID-19 patients: an experience from a low mortality country. Health Sci Rep.

[18] Zhou X.D., Chen Q.F., Targher G. (2024). Global burden of disease attributable to metabolic risk factors in adolescents and young adults aged 15–39, 1990–2021. Clin Nutr.

[19] Ministry of Public Health Qatar (2023). Health Risk Prevalence Data (STEPwise Survey 2023). https://www.data.gov.qa/explore/dataset/health-risk-prevalence-data-stepwise-survey-2023/information/.

[20] Al-Kuwari M.G., Al-Abdulla S.A., Abdulla M.Y. (2021). Epidemiological health assessment in primary healthcare in the State of Qatar-2019. Qatar Med J.

[21] Murray C.J.L. (2022). The Global Burden of Disease Study at 30 years. Nat Med.

[22] Al-Thani M., Al-Thani A., Alyafei S. (2018). The prevalence and characteristics of overweight and obesity among students in Qatar. Public Health.

[23] NCD Risk Factor Collaboration NCD-RisC (2024). Worldwide trends in underweight and obesity from 1990 to 2022: a pooled analysis of 3663 population-representative studies with 222 million children, adolescents, and adults. Lancet.

[24] Abulkhair T., Consunji R., El-Menyar A., Chichaya T.F., Asim M., Al-Thani H. (2025). Trend of injury severity and road traffic-related mortality in an Arab Middle Eastern Country: a 12-year retrospective observational study. Healthcare.

[25] Tarlochan F., Ibrahim M.I.M., Gaben B.J. (2022). Understanding traffic accidents among young drivers in Qatar. Int J Environ Res Public Health.

[26] Mabry R.M., Reeves M.M., Eakin E.G., Owen N. (2010). Gender differences in prevalence of the metabolic syndrome in gulf cooperation council countries: a systematic review. Diabet Med.

[27] Moradi-Lakeh M., Toumi A., Khalifa S.E. (2023). Core health indicators in countries with high proportion of expatriates: case study of Qatar. Front Public Health.

[28] Mehmood A., Maung Z., Consunji R.J. (2018). Work related injuries in Qatar: a framework for prevention and control. J Occup Med Toxicol.

[29] GBD 2023 Demographics Collaborators (2025). Global age-sex-specific all-cause mortality and life expectancy estimates for 204 countries and territories and 660 subnational locations, 1950-2023: a demographic analysis for the Global Burden of Disease Study 2023. Lancet.

