# Supplementary material for: Population health outcomes in Qatar 1990–2023: a systematic analysis for the Global Burden of Disease Study 2023
Source: eClinicalMedicine. 2026 May 18;95:103922. doi: 10.1016/j.eclinm.2026.103922 (PMC13208085; doi:10.1016/j.eclinm.2026.103922)
Supplement: Supplementary Captions [file mmc7.docx]

**Captions for Supplementary Material**

**Manuscript ID:** eclinm-D-26-00671

**Title:** *Population health outcomes in Qatar 1990–2023: a systematic analysis for the Global Burden of Disease Study 2023*

**Journal:** eClinicalMedicine

**Supplementary Figures**

**Supplementary Figure S1.** Age-standardised DALY rates per 100,000 attributable to Level 2 risk factors, Qatar, 1990 and 2023. This bump chart displays the ranking of 20 Level 2 risk factors by age-standardised disability-adjusted life year (DALY) rates per 100,000 population in Qatar in 1990 (left) and 2023 (right). Connecting lines trace the change in ranking position for each risk factor over the 33-year study period. Risk factors are colour-coded by category: metabolic risks (purple), environmental and occupational risks (green), and behavioural risks (orange). The chart illustrates the transition from a risk profile dominated by malnutrition and air pollution in 1990 to one led by metabolic risk factors—particularly high body-mass index, high fasting plasma glucose, and dietary risks—by 2023, consistent with Qatar’s epidemiological and nutritional transition.

**Supplementary Tables**

**Supplementary Table S1.** All-age mortality estimates, percent change, and annualised rate of change for all causes in Qatar (1990–2023). This table presents the absolute number of deaths (both sexes, all ages) with 95% uncertainty intervals (UI) for all GBD 2023 cause categories in Qatar at four time points (1990, 2000, 2015, and 2023). Percentage change (PC) and annualised rate of change (ARC) are reported for three periods: pre-Millennium Development Goals (1990–2000), MDG era (2000–2015), early Sustainable Development Goals era (2015–2023), and the full study period (1990–2023). Causes follow the GBD four-level hierarchical classification. Uncertainty intervals are derived from 250 posterior draws.

**Supplementary Table S2.** All-age prevalence estimates, percent change, and annualised rate of change for all causes in Qatar (1990–2023). This table presents the absolute number of prevalent cases (both sexes, all ages) with 95% uncertainty intervals (UI) for all GBD 2023 cause categories in Qatar at four time points (1990, 2000, 2015, and 2023). Percentage change and annualised rate of change are reported for the same four periods described in Supplementary Table S1. Changes in absolute prevalence should be interpreted in the context of Qatar’s more than sixfold population growth over the study period.

**Supplementary Table S3.** All-age incidence estimates, percent change, and annualised rate of change for all causes in Qatar (1990–2023). This table presents the absolute number of incident cases (both sexes, all ages) with 95% uncertainty intervals (UI) for all GBD 2023 cause categories in Qatar at four time points (1990, 2000, 2015, and 2023). Percentage change and annualised rate of change are reported for the same four periods described in Supplementary Table S1. Changes in absolute incidence should be interpreted in the context of Qatar’s more than sixfold population growth over the study period.

**Supplementary Appendices**

**Supplementary Appendix S1.** Guidelines for Accurate and Transparent Health Estimates Reporting (GATHER). Completed GATHER checklist indicating where each of the 18 reporting items is addressed in the manuscript, with corresponding page numbers. The GATHER statement provides a framework for transparent reporting of global health estimates.

**Supplementary Appendix S2.** Definitions of GBD metrics. This appendix provides definitions of the principal epidemiological metrics used in the GBD 2023 framework, including disability-adjusted life years (DALYs), years of life lost (YLLs), years lived with disability (YLDs), incidence, prevalence, and age-standardised rates, along with the GBD cause and risk factor hierarchical classification system.

**Supplementary Appendix S3.** Full GBD cause hierarchy. This appendix presents the complete four-level GBD 2023 cause hierarchy used in this study, comprising 3 Level 1 cause groups, 22 Level 2 causes, 174 Level 3 causes, and 301 Level 4 causes. The hierarchy classifies all causes of death and disability into communicable, maternal, neonatal, and nutritional (CMNN) diseases; non-communicable diseases (NCDs); and injuries.
