# Supplementary figures and images for: Population health outcomes in Qatar 1990–2023: a systematic analysis for the Global Burden of Disease Study 2023
Source: eClinicalMedicine. 2026 May 18;95:103922. doi: 10.1016/j.eclinm.2026.103922 (PMC13208085; doi:10.1016/j.eclinm.2026.103922)

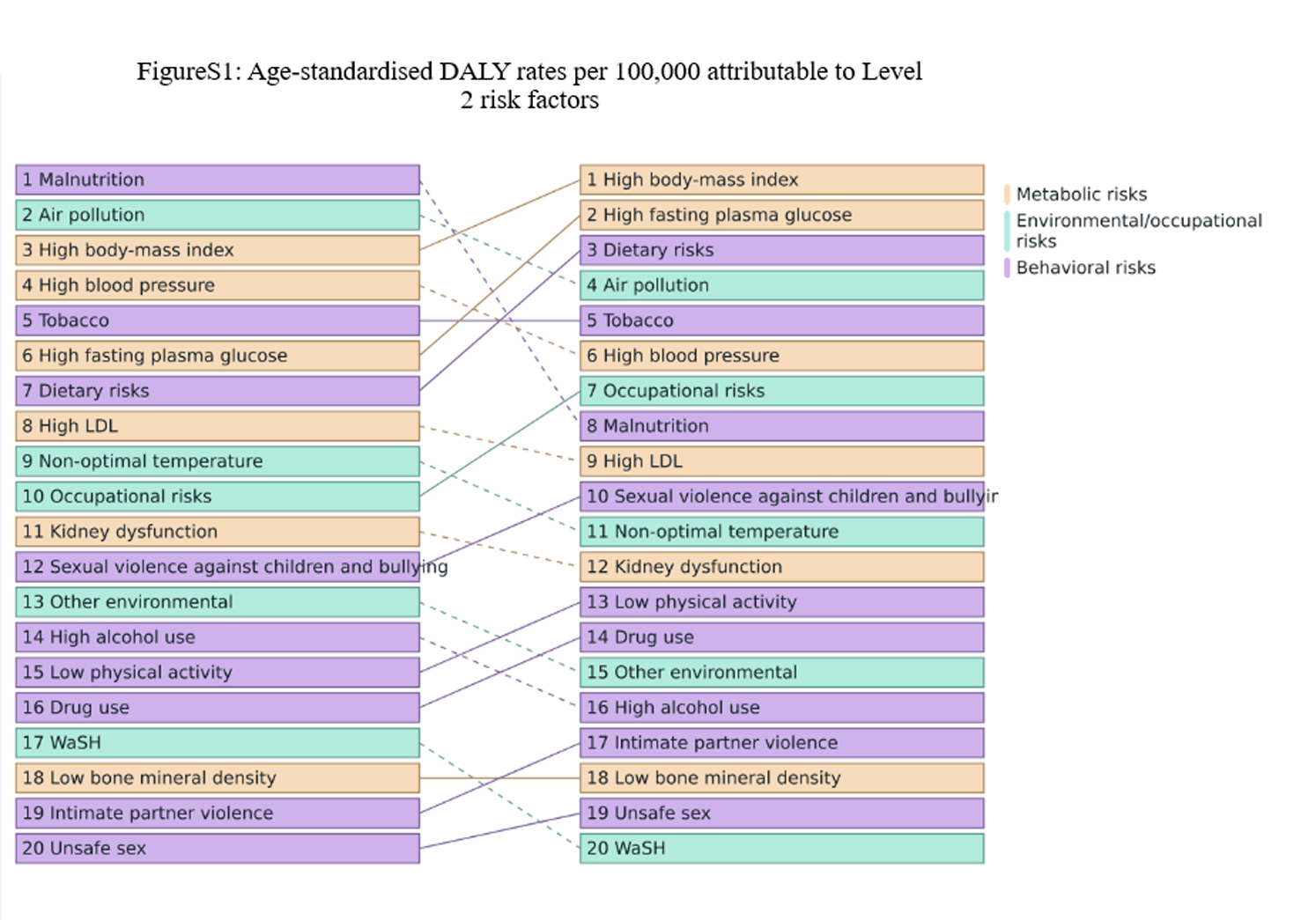

Supplement: Supplementary Figure [file figs1.jpg]
